# Heat stress-induced degradation of glutamine synthetase rebalances central carbon-nitrogen metabolism and promotes thermotolerance in *Ganoderma lucidum*

**DOI:** 10.1128/aem.00602-26

**Published:** 2026-07-21

**Authors:** Jinjin Qiao, Huajun Li, Yuzhen Yang, Yanqiu Li, Shangshang Zhang, Mingwen Zhao, Jing Zhu

**Affiliations:** 1 Department of Microbiology, College of Life Sciences, Nanjing Agricultural University 98430, Nanjing, Jiangsu, People's Republic of China; The University of Arizona, Tucson, Arizona, USA

**Keywords:** heat stress, glutamine synthetase, ubiquitin-proteasome system (UPS), *Ganoderma lucidum*

## Abstract

Thermotolerance is fundamental to fungal ecology and survival. Although heat stress triggers extensive metabolic reprogramming, the function of these changes for thermotolerance has remained poorly understood. Here, we identify glutamine synthetase (GS), a central nitrogen metabolism enzyme, as a critical determinant of thermotolerance in *Ganoderma lucidum*. Silencing of *gs* significantly enhanced fungal tolerance under heat stress and reduced the relative inhibition rate of mycelial growth to 9.71%, compared with 20.7% in the wild-type (WT) strain. Heat stress also increased reactive oxygen species and H_₂_O_₂_ levels by 1.49- and 1.38-fold in the WT strain, whereas the increments were markedly lower in *gs*-silenced strains. Subsequently, under heat stress, α-ketoglutarate contents in WT increased by 1.49-fold. Inhibition of GS further increased the accumulation, which was achieved by upregulating glutamate dehydrogenase to promote the conversion of glutamate to α-ketoglutarate. This metabolic response was correlated with the generation of adenosine triphosphate (ATP) and nicotinamide adenine dinucleotide (NADH). Moreover, under heat stress, the level of GS protein in WT strains decreased by 29.0%, compared with that under normal conditions, due to accelerated degradation via the 26S proteasome. Our findings reveal that a ubiquitin-dependent signal instantaneously rebalances central carbon-nitrogen metabolism, offering a direct and rapid link between central metabolism and thermotolerance in fungi.

**IMPORTANCE:**

Understanding how organisms adapt to heat stress is of increasing urgency in the context of global warming. While the roles of heat-shock proteins and antioxidant systems are well established, how microbes actively reprogram central metabolism to survive thermal challenge remains a fundamental, unanswered question. This study reveals that the central nitrogen metabolism enzyme glutamine synthetase (GS) is degraded by the ubiquitin-proteasome system and that this degradation acts as a metabolic switch to enhance thermotolerance in *Ganoderma lucidum*. We discovered that heat stress induces ubiquitin-proteasome system-dependent GS degradation, leading to redirected central nitrogen flux that elevates α-ketoglutarate content. This metabolic shift boosts ATP and NADH production. In summary, our findings represent a significant advance beyond classical protein chaperone systems and reactive oxygen species-scavenging systems, highlighting a direct and rapid link between metabolic flux and thermotolerance.

## INTRODUCTION

Microbial growth is tightly tied to ambient temperature, yet cells cannot control their internal heat, making temperature fluctuation an unavoidable stress (1). As a fundamental environmental variable, heat stress dictates the growth, metabolism, and ecological distribution of microbes (2). Once under heat stress, membrane integrity is compromised, protein folding is perturbed, and a burst of reactive oxygen species (ROS) ensues, ultimately causing nutrient loss, cytotoxicity, and programmed cell death (3, 4). Most studies have focused on the heat stress functions of the resulting signals, master regulators, heat-shock proteins, and antioxidant defense systems (5). Membrane fluidity, MAPK pathway, and Ca²^+^ and NO signals change when microbes suffer heat stress (6–8). These signaling pathways can activate master regulators, such as heat shock transcription factors and the NAC and MYB families (9, 10). The transcriptional switch initiates heat-shock proteins to stabilize folding intermediates, drives the accumulation of trehalose and glutathione to scavenge ROS, and elevates antioxidant enzymes to detoxify the radicals generated by heat stress (11–13).

Despite those mechanistic insights, a unifying principle emerges, in which heat stress elicits central metabolic rewiring. Multi-omics studies have revealed that in taxonomically diverse microbes, including *Escherichia coli*, *Aspergillus niger*, *Candida* spp., *Lentinula edodes,* and *Ganoderma lucidum*, heat stress rewires glycolytic flux and pyruvate fate, coinciding with alterations of tricarboxylic acid cycle intermediates, amino acid pools, and polyol biosynthesis (14–18). The functional benefits of these metabolic alterations are beginning to be elucidated. For instance, in *Metarhizium robertsii*, heat stress resulted in accumulation of pyruvate, which scavenges ROS and stabilizes mitochondrial membranes (19). Heat stress activates the pentose-phosphate pathway to produce more nicotinamide adenine dinucleotide phosphate (NADPH) and glutathione (GSH), thereby alleviating heat stress in *G. lucidum* and *Pleurotus ostreatus* (20, 21). However, the mechanisms through which heat stress triggers metabolic rewiring and the functional contribution of this reprogramming to thermotolerance remain poorly understood.

Glutamate and glutamine serve as nitrogen donors for the biosynthesis of all nitrogen-containing compounds in the cell. The interconversion among α-ketoglutarate, glutamate, and glutamine is recognized as central nitrogen metabolism (CNM) (22). Glutamine synthetase (GS), a key enzyme of CNM, catalyzes the ATP-dependent formation of glutamine from glutamate and ammonia. Beyond its canonical role in nitrogen metabolism, GS also participates in growth and development, secondary metabolism, and virulence in fungi (23–25). Emerging evidence suggests that GS additionally regulates carbon/nitrogen metabolic balance and stress responses (26). Recent studies in *Enterobacteriaceae* revealed that the expression of 2-oxoglutarate dehydrogenase, the rate-limiting enzyme of the TCA cycle, is repressed at the post-transcriptional level by the small RNA GlnZ, which is derived from the 3′ UTR of the GS-encoding glnA mRNA (27). In mammals, GS is frequently upregulated in various cancer cells and is closely associated with tumorigenesis by fueling the TCA cycle to support rapid cell proliferation (28). In plants, GS is known to respond to a range of drought, low temperature, and salinity (29). In contrast, in fungi, aside from its well-established role, the additional physiological and regulatory functions of GS remain largely unexplored.

*G. lucidum*, a large basidiomycete fungus, has a considerable market in Southeast Asia due to its diverse bioactive metabolites. With the completion of genome sequencing and the development of transgenic systems, it has become a model organism for investigating molecular regulation in large basidiomycetes. Our previous studies linked thermotolerance to remodeling of membrane fluidity, NO and Ca^2+^ homeostasis, and AMP-activated protein kinase signaling (6, 21, 30–33). Additionally, transcriptomic profiling analyses have revealed that heat stress induces significant changes in metabolism-related genes in *G. lucidum* (34). Further studies revealed that NO alleviates heat stress-induced damage by inhibiting aconitase activity and reducing mitochondrial ROS levels (30). Meanwhile, the enhanced glycolytic pathway produces more NADPH to combat the sustained increase in intracellular ROS (21). However, the role of central nitrogen metabolism in thermotolerance remains unclear, and the underlying mechanisms are poorly understood.

Here, we targeted degradation of glutamine synthetase via the ubiquitin-proteasome system (UPS), which acts as a metabolic switch that actively promotes thermotolerance in *G. lucidum*. We discovered that heat stress induces UPS-dependent GS degradation, leading to redirected nitrogen flux that elevates α-ketoglutarate content. This metabolic shift boosts ATP and NADH production while reducing reactive oxygen species accumulation. Our findings reveal a ubiquitin-dependent control of GS activity that rebalances central carbon-nitrogen metabolism to sustain growth under thermal challenge.

## MATERIALS AND METHODS

### Strains and culture conditions

The *Ganoderma lucidum* strain (ACCC53264) was provided by the Agricultural Culture Collection of China. The wild-type (WT) and empty carrier control (CK) strains were used as controls, and two *gs*-silenced strains (*gs*i-5 and *gs*i-6) were constructed in our previous study (24). In this study, two *gdh*-silenced strains (*gdh*i-5 and *gdh*i-12) and two *gs-gdh* co-silenced strains (*gs-gdh*i-20 and *gs-gdh*i-22) were constructed and screened. WT, CK, and various silenced strains were cultured in complete yeast medium (CYM), containing 20 g/L glucose, 4.6 g/L KH_2_PO_4_, 0.5 g/L MgSO_4_·7H_2_O, 2 g/L tryptone, and 2 g/L yeast extract. Strains were inoculated onto CYM medium and grown at 28°C for 5 days, followed by heat stress treatment at 42°C for 30 min. Then, the plate was immediately returned to 28°C and incubated for 2 days. The relative inhibition rate of different strains under non-heat and heat stress was calculated as (Dc − Dt)/Dc × 100 (%), where Dc and Dt represent the colony diameter without heat stress and the colony diameter with heat stress, respectively. *Escherichia coli* DH5α (Sangon Biotech, Shanghai, China) was cultured in Luria-Bertani medium containing ampicillin (100 µg·mL^−1^) or kanamycin (50 µg·mL⁻¹) for extracting the constructed plasmid.

### Construction of silenced strains

The RNA interference method was used to construct the strains with silenced glutamate dehydrogenase (*gdh*) and the strains with co-silenced glutamine synthetase and glutamate dehydrogenase (*gs-gdh*). The primers for the silenced sequences of *gdh* and *gs-gdh* are shown in Table S1, using cDNA from *G. lucidum* as the template. The amplified fragments were ligated to the previously constructed pAN7-ura3-dual vector (6). Finally, the pAN7-dual-*gdh* and pAN7-dual-*gs-gdh* vectors were obtained. These vectors were transformed into the WT strain via *Agrobacterium*-mediated transformation (6). For selection of transformants, 250 µg·mL⁻¹ hygromycin B was used. Two independent high-efficiency knockdown strains for each gene were selected for further experiments, as shown in Fig. S2. The WT strain transformed with the carrier plasmid (pAN7-*ura3*-dual-*hyg*) was used as the empty-vector control.

### Quantitative real-time PCR analysis of gene expression

Total RNA from the samples was extracted using RNAiso Plus reagent (Vazyme, Nanjing, China) and then reverse transcribed into cDNA using the Hiscript III RT SuperMix (Vazyme, Nanjing, China). Using the primers listed in Table S1, with the cDNA of *G. lucidum* as the template and SYBR Green qPCR SuperMix reagent (Vazyme, Nanjing, China), gene expression levels were detected by quantitative real-time PCR. Using 18S rRNA of *G. lucidum* as the housekeeping gene, the relative expression level of the target gene was calculated by the 2^−∆∆CT^ method (35).

### Assay of enzyme activity and substance content

The frozen hyphae (0.1 g) were ground into powder using liquid nitrogen and mixed with 1 mL of crude protein extraction solution (0.5 M Tris-HCl, 10% sodium dodecyl sulfate [SDS], 20% glycerin, 1 M dithiothreitol, and 100 mM phenylmethyl sulfonyl fluoride). Then, centrifugation was performed at 12,000 × *g* for 15 min at 4°C. The supernatant was collected and subsequently used for detecting enzyme activity and substance content.

The activities of glutamine synthetase, glutamate dehydrogenase (GDH), glutamate synthase (GOGAT), citrate synthase (CS), isocitrate dehydrogenase (ICDH), and α-ketoglutarate dehydrogenase (α-KGDH) were measured using corresponding activity assay kits (Solarbio, Beijing, China), following the manufacturer’s instructions. The content of H_2_O_2_, ATP, and NADH was also measured using corresponding assay kits (Solarbio, Beijing, China), following the manufacturer’s instructions.

### Detection of intracellular ROS levels

The measurement of intracellular ROS levels was performed using the method previously described (36). First, sterile cover slips were inserted at an angle into the bottom of the culture medium. Once the mycelia had grown on the cover slips, they were stained with 2′,7′-dichlorodihydro-fluorescein diacetate (DCFH-DA) (Solarbio, Beijing, China) for 30 min, followed by washing away the excess dye. The fluorescence was detected using a Zeiss Axio Imager A1 fluorescence microscope (Zeiss, Oberkochen, Germany). For each treatment, 20 pieces of photos were selected for fluorescence intensity. The average fluorescence intensity was analyzed using ZEN Lite software (Zeiss, Oberkochen, Germany). The fluorescence level of the WT strain under non-heat stress was set to 1, and the ROS fluorescence intensity of various strains under both non-heat and heat stress was compared.

### Western blot analysis

For the extraction of total protein, fresh mycelia (0.1 g) were ground into powder using liquid nitrogen, followed by the addition of 800 µL of extraction buffer (0.5 M Tris-HCl, 10% SDS, 20% glycerol, 1 M dithiothreitol, and 100 mM phenylmethyl sulfonyl fluoride). The resulting homogenate was then chilled on ice for 10 min prior to centrifugation at 10,000 × *g* for 10 min. The supernatant was collected, and protein concentration was assessed according to the Bradford method. The protein content was detected by Western blotting according to the methods described previously (24, 37). The primary antibodies were a β-actin antibody (Engibody, Beijing, China) with a dilution of 1:1,500 and a GS antibody with a dilution of 1:1,000, which was prepared in our previous study (24). The secondary antibodies were HRP goat anti-Rabbit IgG antibody (Engibody, Beijing, China) with a dilution of 1: 5,000. The signals were finally detected using the ECL Western blotting detection kit (Vazyme, Nanjing, China) and the chemiluminescence detection system (Bio-Rad, Hercules, CA, USA).

### Cell-free protein degradation assay

The mycelia were ground into powder using liquid nitrogen. Then, 0.1 g of this powder was transferred into 800 µL of soluble crude extract solution containing 25 mM Tris-HCl at pH 7.5, 10 mM NaCl, 10 mM MgCl_2_, 5 mM dithiothreitol, and 1 mM phenylmethyl sulfonyl fluoride. The mixture was placed on ice for 15 min before being centrifuged at 12,000 × *g* for 15 min. The resulting supernatant was collected for further analysis. Subsequently, 200 µL of the purified GS-His recombinant protein was added to 200 µL of the extract and incubated at 30°C for 0, 3, 6, 9, and 12 h, respectively, in the presence of 10 mM ATP. Before incubation, 50 μM Carbobenzoxy-l-leucyl-l-leucyl-l-leucinal (MG132) (Solarbio, Beijing, China) or 20 μM Aloxistatin (E64d) (Aladdin, Shanghai, China) was added. Dimethyl sulfoxide (DMSO) was added as a negative control. In these treated samples, 5× protein loading sample buffer (5 mM Tris-HCl at pH 6.8, 2% [wt/vol] SDS, 10% glycerol, 2.5% [vol/vol] β-mercaptoethanol, and 0.05% [wt/vol] bromophenol blue) was added, and the mixture was boiled for 10 min to terminate the reaction. Finally, the content of the GS protein was assessed using WB with anti-His antibody (Abcam, Shanghai, China), GS antibody, and β-actin antibody, respectively.

### Immunoprecipitation and ubiquitination assays *in vivo*

Fresh samples, both non-heat stress and heat stress, were ground in liquid nitrogen. NP-40 lysis buffer (50 mM Tris, 150 mM NaCl, 1 mM EDTA, and 1% NP-40 [pH 7.4]) was added to the samples as required by the instructions of the Protein A/G Magnetic Beads kit (Vazyme, Nanjing, China). Protease inhibitor (cocktail and MG132) was added to prevent protein degradation. After mixing, the samples were kept on ice for 20 min, followed by centrifugation at 4°C for 5 min at 13,800 × *g*. The supernatant was collected and kept on ice for further use. A volume of 500 µL of the lysate was incubated with 10 µg of GS antibody. The mixture was gently rotated at 4°C overnight. Protein A/G Magnetic Beads were added to the mixture at a concentration of 50 µL/mL and rotated for another 30 min to capture the immunocomplex. After a brief centrifugation, the mixture was placed on a magnetic stand for 30 s to allow the beads to settle completely. The supernatant was carefully removed, and the beads were washed three times with phosphate-buffered saline. The precipitated samples were resolved by sodium dodecyl sulfate-polyacrylamide gel electrophoresis, and the ubiquitination level of GS was detected using anti-GS antibody and anti-ubiquitin antibody (Abmart, Shanghai, China) with a dilution of 1:1,500.

### Yeast two-hybrid assay

The cDNA library of *G. lucidum* used for yeast two-hybrid (Y2H) screening was constructed in our previous study (38). For screening the interacting proteins of GS, the bait vector pGBKT7-GS was constructed and transformed into the Y2HGold strain. No self-activation was observed. Subsequently, the pGBKT7-GS bait vector and the Y2H library plasmids were co-transformed into the Y2HGold strain. Multiple rounds of selection were performed according to the manufacturer’s instructions to obtain GS-interacting proteins. The Gl24873 protein was selected for further study.

To detect the interaction between GS and Gl24873, the cDNA of *G. lucidum* was used as the template, and the full-length sequences of *gs* and *Gl24873* were obtained by PCR using the primers in Table S1. These sequences were cloned into the pGBKT7 bait vector and the pGADT7 prey vector, respectively. pGBKT7-GS and pGADT7-Gl24873 were transformed into the Y2HGold strain and cultured at 30°C for 2 days on two synthetic dropout media, SD/Leu/−Trp and SD/−Ade/−His/−Leu/−Trp, with 20 ng/mL X-α-gal and 125 ng/mL Aureobasidin A (TaKaRa, Dalian, China). The strain with pGBKT7-53 and pGADT7-T was used as the positive control. The strain with pGBKT7-Lam and pGADT7-T was used as the negative control.

### Bimolecular fluorescence complementation assay

Primers in Table S1 were used to amplify the full-length sequences of *Gl24873* and *gs* by PCR, which were then inserted into pVN and pVC vectors, respectively, for the bimolecular fluorescence complementation (BiFC) assay (39). The recombinant constructs were co-transformed into yeast strain SFY2620 and incubated on SD/−Leu−Ura medium at 30°C for 3 days. Green fluorescence signals were detected by confocal microscopy (Olympus, Japan). The recombinant plasmids PVN plus PVC, PVN-Gl24873 plus PVC, and PVN plus PVC-GS were co-transformed into yeast as negative controls.

### Statistical analysis

The statistical analysis was performed using GraphPad Prism 8.0 (GraphPad Software, Inc., San Diego, CA, USA). All experimental data presented were performed in three independent duplicate samples. The error bar represents the standard deviation from the mean of the three repetitions. Duncan’s multiple range test was used for statistical analysis.

## RESULTS

### Inhibition of GS activity enhances the tolerance of *Ganoderma lucidum* to heat stress

**Fig 1:**
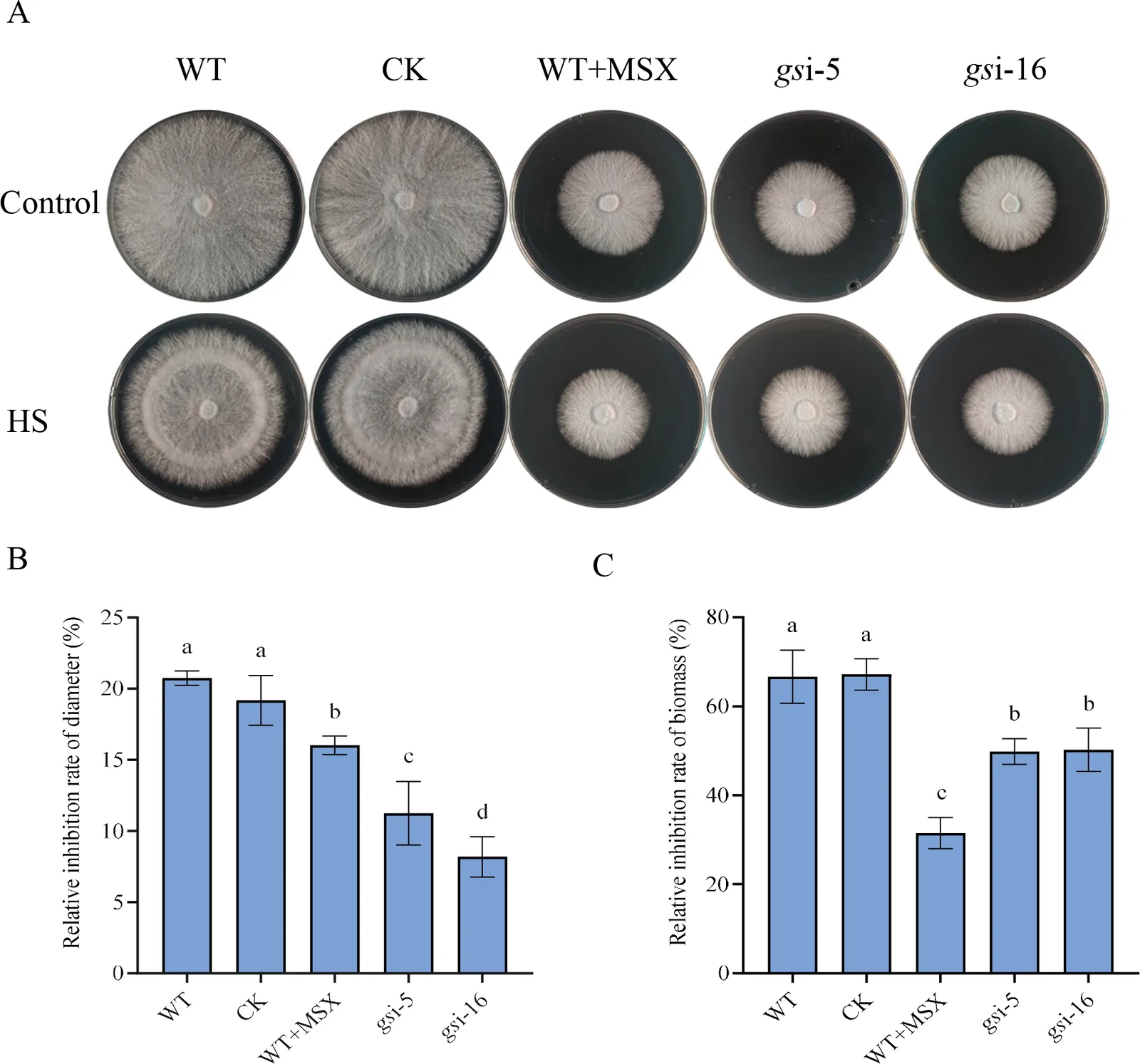
The effect of inhibition of GS activity on mycelial growth of *G. lucidum*. (**A**) Mycelial growth of WT, CK, *gs*-silenced strains, and WT with the addition of inhibitor MSX (2.5 mM) under non-heat and heat stress. (**B**) The relative inhibition rate of different strains under non-heat and heat stress was calculated as (Dc − Dt)/Dc × 100 (%), where Dc and Dt represent the colony diameter under non-heat and heat stress, respectively. (**C**) The relative inhibition rate of biomass of each strain in liquid medium under non-heat stress and heat stress was calculated as (Dc1 − Dt1)/Dc1 × 100 (%), where Dc1 and Dt1 represent the dry weight of mycelia under non-heat and heat stress, respectively. Results are expressed as mean ± SD (*n* = 3). Different letters indicate significant differences between groups (*P* < 0.05, Duncan’s multiple range test).

Our previous studies have revealed that heat stress significantly inhibits mycelial growth, leads to the accumulation of ROS, and causes metabolic rearrangement in *G. lucidum* (6, 21, 34, 40). Glutamine synthetase is a key enzyme in the central nitrogen metabolism of *G. lucidum*; its absence suppresses mycelial growth and secondary metabolite biosynthesis (24). To elucidate the role of central nitrogen metabolism in heat stress defense, we first quantified the impact of GS deficiency on the mycelial growth of the WT strain under heat stress conditions. L-methionine sulfoximine (MSX), an inhibitor of glutamine synthetase (41), was used to treat mycelia. As the MSX concentration increased, colony diameter and GS activity both declined. Compared with the untreated control, supplementation with 2.5 mM MSX reduced mycelial growth by 51.5%, while reducing GS activity by 91.1% (Fig. S1). Relative to WT under normal conditions, heat stress inhibited WT mycelial growth by 20.7%. In contrast, the same stress reduced growth by 16% in WT treated with 2.5 mM MSX, 11.2% in *gs*i-5, and 8.2% in *gs*i-16 (Fig. 1A and B). Compared with normal conditions, heat stress reduced mycelial biomass by 66.7% in WT, 67.2% in CK, 31.5% in WT treated with 2.5 mM MSX, 49.8% in *gs*i-5, and 50.2% in *gs*i-16 (Fig. 1C). These results indicate that, under heat stress, reduced GS activity constrains growth yet enhances thermotolerance.

**Fig 2:**
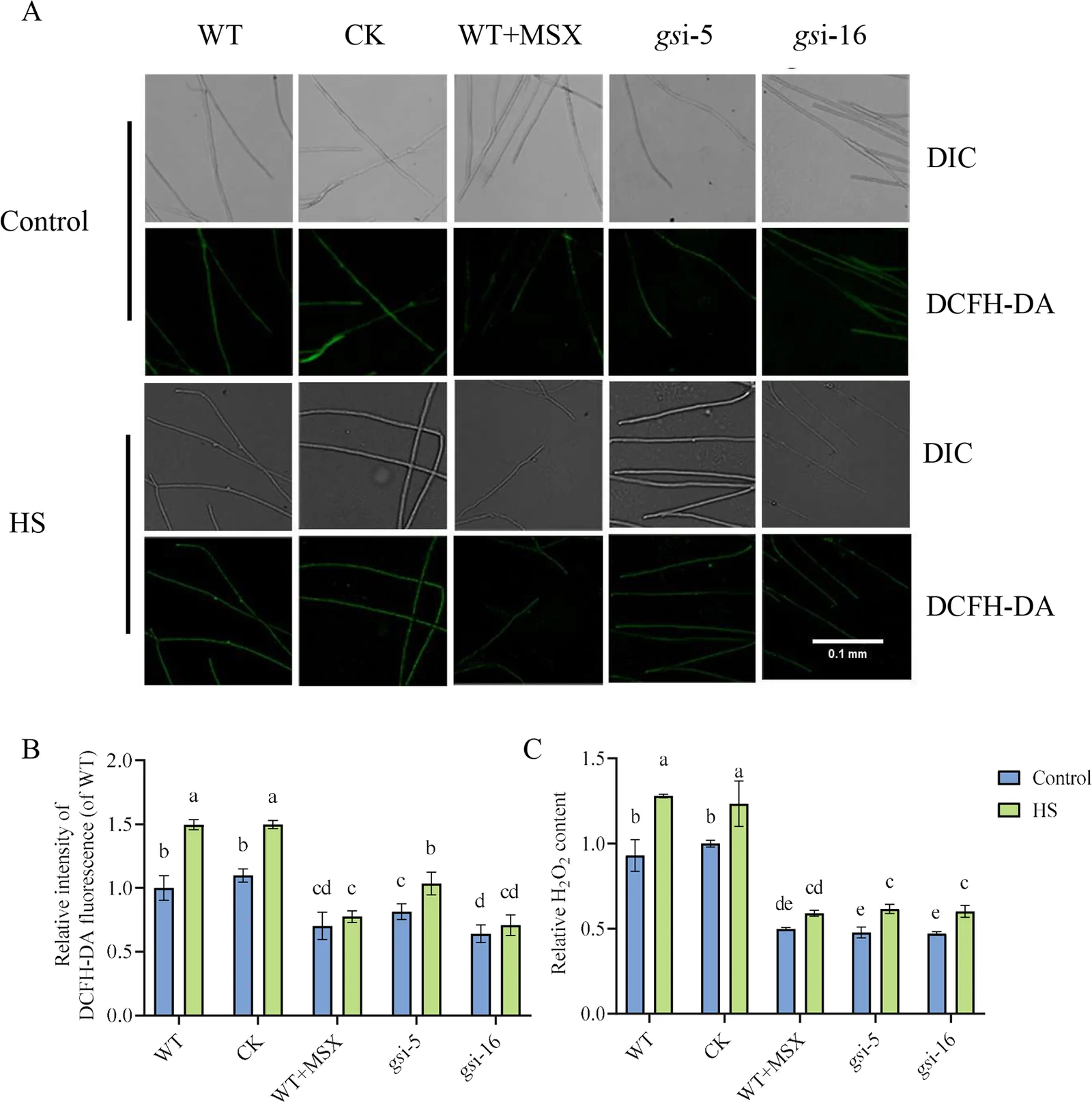
The impact of inhibiting GS activity on the intracellular reactive oxygen species level. (**A**) The intracellular ROS levels in different strains under non-heat and heat stress were detected by DCFH-DA. Scale bar represents 0.1 mm. (**B**) ROS fluorescence ratio of different strains under non-heat and heat stress. (**C**) H_2_O_2_ content of different strains under non-heat and heat stress. The relative H_2_O_2_ content was calculated as the ratio of H_2_O_2_ content of different strains to that of the WT strain under non-heat stress. The error bar represents the standard deviation of the mean for the three repetitions. Results are expressed as mean ± SD (*n* = 3). Different letters indicate significant differences between groups (*P* < 0.05, Duncan’s multiple range test).

Heat stress typically triggers excessive intracellular accumulation of reactive oxygen species (42). Furthermore, we demonstrate the impact of GS on ROS accumulation under heat stress. Under normal growth conditions, intracellular ROS and H_₂_O_₂_ levels were significantly reduced in MSX-treated WT (by 29.8% and 50.2%, respectively) and in *gs*-silenced strains (by 35.8% and 58.4%), both relative to untreated WT (Fig. 2). Upon heat stress, GS inhibition effectively prevented further ROS accumulation. Compared with WT under normal conditions, heat-stress WT exhibited 1.49- and 1.38-fold increases in ROS and H_₂_O_₂_, whereas *gs*i-5, *gs*i-16, and MSX-treated mycelia showed lower elevations (1.27-, 1.11-, and 1.10-fold for ROS; 1.29-, 1.27-, and 1.18-fold for H_₂_O_₂_) (Fig. 2). These results indicate that GS inhibition substantially mitigates heat-induced intracellular ROS burst.

### Inhibition of GS activity under heat stress increases glutamate and α-ketoglutarate contents

**Fig 3:**
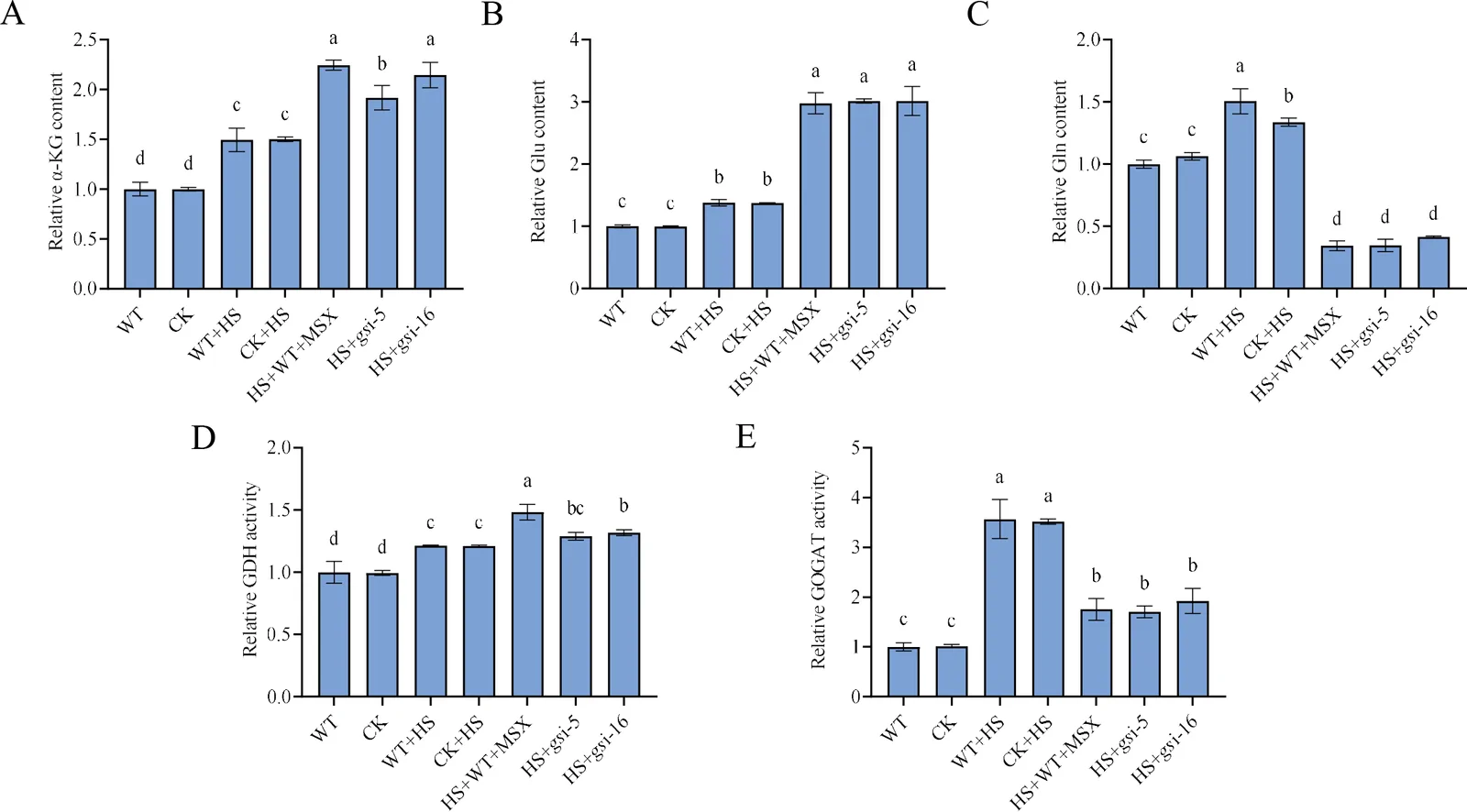
The effects of GS activity on the contents of substances and the activities of enzymes in central nitrogen metabolism. α-ketoglutarate (**A**), glutamate (**B**), and glutamine (**C**) content in WT, CK, and *gs*-silenced strains, and addition of MSX (2.5 mM) in WT under non-heat stress and heat stress. The contents of α-KG, Glu, and Gln in the WT strain under non-heat stress were set to 1. (**D**) The relative enzyme activity of GDH in different strains under non-heat stress and heat stress. (**E**) The relative enzyme activity of GOGAT in different strains under non-heat stress and heat stress. Results are expressed as mean ± SD (*n* = 3). Different letters indicate significant differences between groups (*P* < 0.05, Duncan’s multiple range test).

To elucidate how glutamine synthetase orchestrates central nitrogen metabolism under heat stress, we quantified intracellular α-ketoglutarate (α-KG), glutamate, and glutamine contents in WT, MSX-treated WT, and *gs*-silenced strains (*gs*i-5 and *gs*i-16) (Fig. 3A through C). Compared with normal conditions, under heat stress, the contents of α-KG, glutamate, and glutamine in the WT strain increased significantly by 1.45-, 1.38-, and 1.51-fold, respectively. Under heat stress, compared with WT, the contents of glutamate in the MSX-treated WT, *gs*i-5, and *gs*i-16 strains further accumulated by 2.16-, 2.18-, and 2.18-fold, respectively (Fig. 3B). The contents of α-KG also further increased by 1.50-, 1.28-, and 1.43-fold, respectively (Fig. 3A). However, glutamine decreased by 77.0%, 72.5%, and 77.2% in *gs*i-5, *gs*i-16, and MSX-treated WT strains, respectively (Fig. 3C). These results indicate that heat stress elevates the contents of glutamate, α-KG, and glutamine, while the inhibition of glutamine synthetase amplifies the accumulation of glutamate and α-KG.

Furthermore, we monitored the activities of glutamate dehydrogenase and glutamate synthase in the central nitrogen metabolism (Fig. 3D and E). Compared with normal conditions, under heat stress, the activities of GDH and GOGAT in the WT strain increased significantly by 1.21- and 3.57-fold, respectively. Under heat stress, compared with WT, the activity of GDH increased significantly by 1.22-fold after GS activity was inhibited (Fig. 3D). In contrast, the activity of GOGAT decreased significantly by 50.8% after GS activity was inhibited (Fig. 3E). Collectively, GS inhibition affects central nitrogen metabolism under heat stress by enhancing GDH activity.

### Inhibition of GS activity under heat stress increases α-KG accumulation through GDH

**Fig 4:**
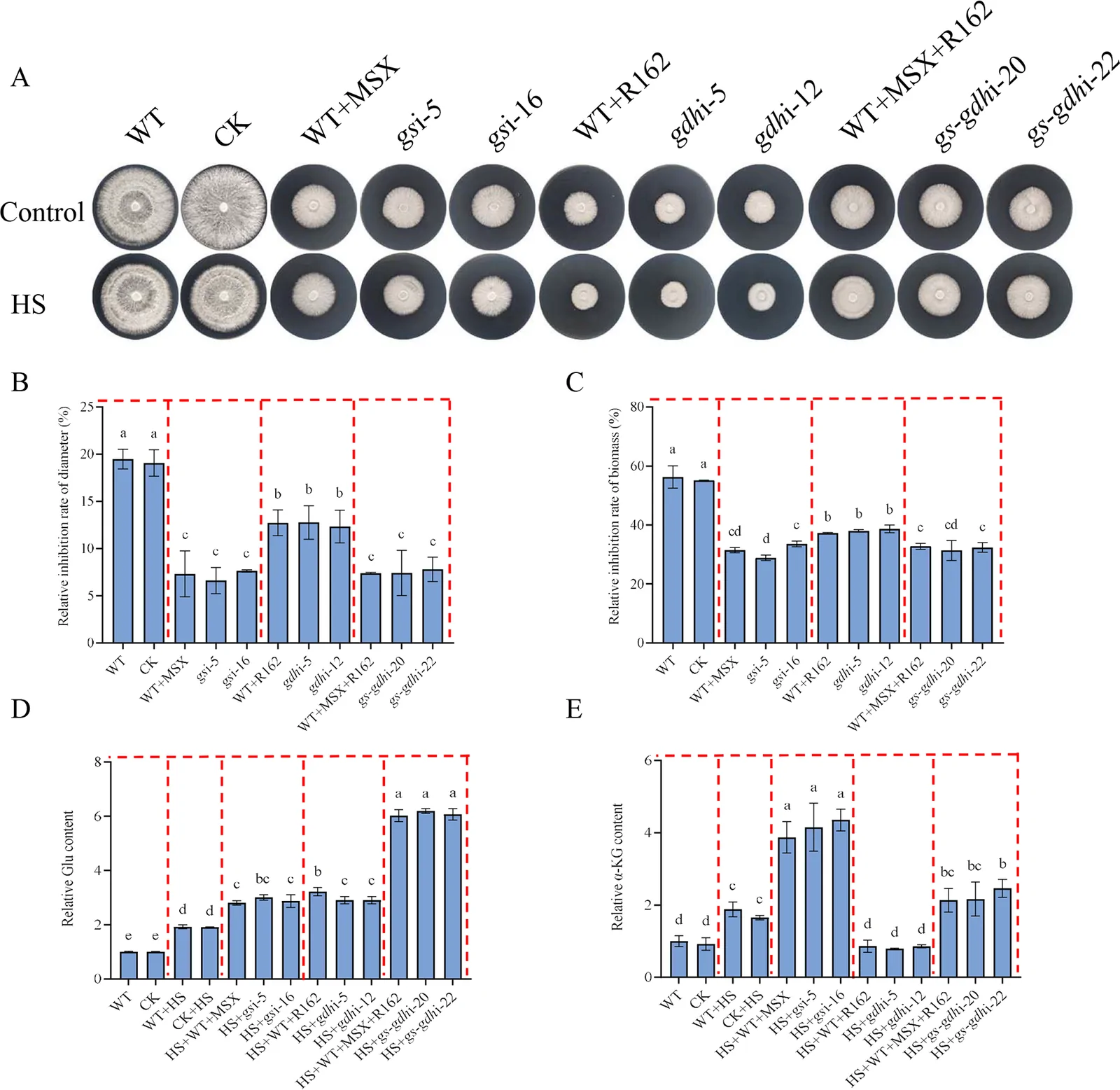
The effect of GS and GDH activities on mycelial growth and central nitrogen metabolism. (**A**) Mycelial growth of WT, CK, *gs*-silenced strains, *gdh*-silenced strains, *gs-gdh* co-silenced strains, strains treated with the inhibitor MSX (2.5 mM) or R162 (0.02 mg mL^−1^) alone, and strains treated with both inhibitors MSX (2.5 mM) and R162 (0.02 mg mL^−1^) under non-heat and heat stress. (**B**) The relative inhibition rate of different strains under non-heat and heat stress was calculated as (Dc − Dt)/Dc × 100 (%), where Dc and Dt represent the mycelial diameter under non-heat and heat stress, respectively. (**C**) The relative inhibition rate of biomass of each strain in liquid medium under non-heat and heat stress was calculated as (Dc1 − Dt1)/Dc1 × 100 (%), where Dc1 and Dt1 represent the dry weight of mycelia under non-heat and heat stress, respectively. The relative content of Glu (**D**) and α-KG (**E**) in different strains under heat and non-heat stress. The contents of Glu and α-KG in the WT strain without heat stress were set to 1. Results are expressed as mean ± SD (*n* = 3). Different letters indicate significant differences between lines (*P* < 0.05, Duncan’s multiple range test).

To dissect the contribution of enhancing GDH activity triggered by *gs* silencing under heat stress, we established experimental materials that allowed independent or simultaneous inhibition of GDH and GS activities. First, the WT strain was treated with 1-hydroxy-2-(2-propen-1-yl)-9,10-anthracenedione (R162), a selective GDH inhibitor. Compared with normal conditions, supplementation with 0.02 mg mL⁻¹ R162 markedly suppressed mycelial growth and reduced GDH activity by approximately 30.5% (Fig. S2A through C). Next, three independent *gdh*-silenced strains (*gdh*i-5, *gdh*i-11, and *gdh*i-12) were generated, in which *gdh* expression was downregulated by approximately 70% (Fig. S2D). We further constructed co-silenced strains of *gs* and *gdh*; three transformants (*gs-gdh*i-8, *gs-gdh*i-20, and *gs-gdh*i-22) with 60%–70% silencing efficiency were selected for subsequent analyses (Fig. S2E). In parallel, MSX and R162 were co-applied to WT mycelia to achieve simultaneous enzymatic inhibition of GS and GDH. After heat stress, WT mycelia exhibited 19.5% and 56.2% inhibition in radial growth and biomass, respectively (Fig. 4A through C). In contrast, *gs*-silenced strains showed significantly lower inhibition (7.12% and 31.2%). Single inhibition of GDH, via either *gdh* silencing or R162 treatment, resulted in inhibition of 12.6% and 38.0%, respectively. Importantly, additional suppression of GDH under a GS-deficient background (co-silenced strains or MSX + R162 co-treatment) did not further alter these parameters.

Furthermore, the glutamate and α-KG contents revealed pronounced perturbations under heat stress. Under heat stress, compared with WT, intracellular glutamate in *gdh*i-5, *gdh*i-12, and R162-treated WT increased 1.51-, 1.51-, and 1.67-fold, respectively, whereas α-KG decreased by 58.0%, 54.4%, and 54.2% (Fig. 4D and E), confirming that GDH deficiency leads to glutamate accumulation and α-KG decrease. When both GS and GDH were suppressed, glutamate levels in *gs-gdh*i-20, *gs-gdh*i-22, and MSX + R162-treated WT rose to 3.22-, 3.15-, and 3.13-fold of WT, respectively. Notably, under heat stress, compared with the WT strain, the content of α-KG in the double-silenced strains showed no significant change, but it was significantly reduced compared with the *gs*-silenced strains, with a decrease of 45.43%. Compared with the *gdh*-silenced strains, it was significantly increased, with an increase of 2.70-fold. These data indicate that *gs* silencing induced GDH activity to generate α-KG.

### α-KG accumulation enhances the thermotolerance of mycelia

**Fig 5:**
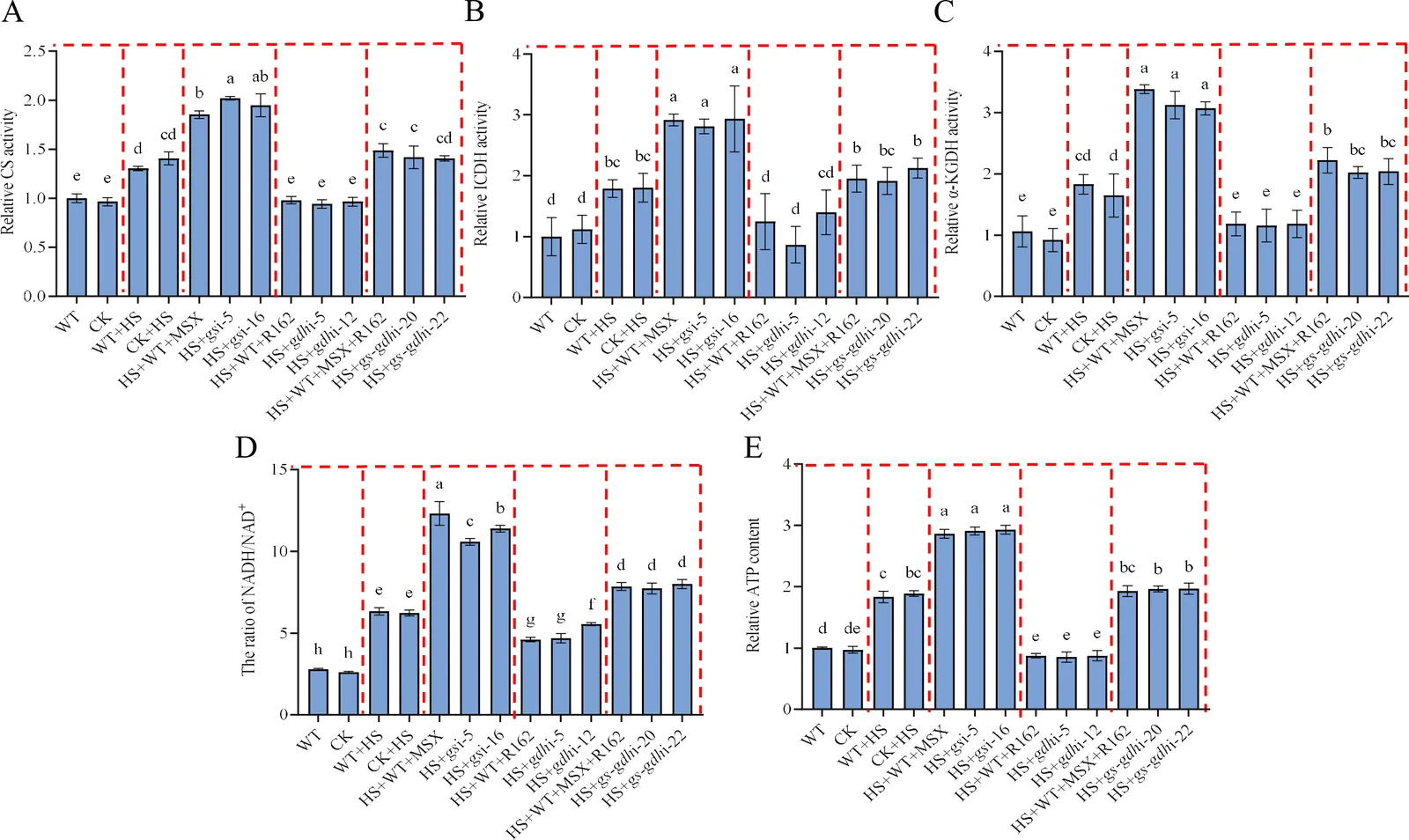
The effects of GS and GDH activities on the activity of key enzymes in the TCA cycle, ATP content, and the NADH/NAD^+^ ratio. (**A**) Relative enzyme activity of CS under non-heat stress and heat stress in WT, CK, *gs*-silenced strains, *gdh*-silenced strains, *gs-gdh* double-silenced strains, strains with the addition of inhibitor MSX (2.5 mM) or R162 (0.02 mg mL^−1^) alone, and strains with the addition of inhibitors MSX (2.5 mM) and R162 (0.02 mg mL^−1^). (**B**) Relative enzyme activity of ICDH. (**C**) Relative enzyme activity of α-KGDH. (**D**) Ratio of NADH/NAD^+^, and (**E**) relative content of ATP. The enzyme activity of CS, ICDH, and α-KGDH and the contents of ATP in the WT strain without heat stress were set to 1. The results are expressed as mean ± standard deviation (*n* = 3). Different letters indicate significant differences between groups (*P* < 0.05, Duncan’s multiple range test).

α-KG is an important connection point between central carbon and nitrogen metabolism (43). We suggested that heat stress-induced α-KG accumulation contributed to the generation of energy and reducing power, thereby defending against heat stress. To test this hypothesis, we detected the activities of key enzymes in the TCA cycle, intracellular ATP content, and the NADH/NAD^+^ ratio under heat stress. Compared with non-stressed conditions, WT under heat stress displayed 1.31-, 1.79-, and 1.73-fold increases in CS, ICDH, and α-KGDH activities, respectively, accompanied by 1.83- and 2.27-fold elevations in ATP and the NADH/NAD^+^ ratio (Fig. 5A through E). *gs*-silenced strains further enhanced these enzyme activities. The activities of CS, ICDH, and α-KGDH increased 1.55-, 1.57-, and 1.71-fold, respectively, whereas ATP levels and the NADH/NAD^+^ ratio increased by 1.58- and 1.67-fold, respectively, relative to heat-stressed WT. Conversely, GDH suppression alone reduced CS, ICDH, and α-KGDH by approximately 24.9%, 30.2%, and 34.2%, respectively, ATP by 52.3%, and NADH/NAD^+^ by 27.3%. Co-inhibition of GS and GDH restored those enzyme activities and energy status to WT levels. These results indicate that GS inhibition boosted α-KG generation to fuel the TCA cycle and elevated ATP and NADH.

To further validate that α-KG accumulation contributes to mycelial thermotolerance, we supplemented the WT strain with various concentrations of α-KG (0–150 mg L^−1^) under both non-heat-stress and heat-stress conditions, respectively. As shown in Fig. S4, compared with the untreated control (0 nmol/g; inhibition rate of 17.4%), the 60 mg L^−1^ treatment group exhibited the lowest relative inhibition rate (8.6%). Furthermore, we assessed mycelial growth and H_₂_O_₂_ content in different strains following 60 mg L^−1^ α-KG supplementation under non-heat-stress and heat-stress conditions (Fig. S5A through C). Compared with the untreated WT (inhibition rate of 20.3%), the relative inhibition rates of mycelial diameter were significantly reduced in both the wild-type and various mutant strains after exogenous α-KG application, reaching 12.0%, 11.9%, 10.4%, 9.7%, 8.4%, and 8.1%, respectively. Relative to normal conditions, H_₂_O_₂_ content in the heat stress wild-type strain increased by 1.91-fold, whereas α-KG-supplemented WT showed a 1.51-fold increase. In the *gdh*-silenced strain, H_₂_O_₂_ levels increased by 1.70- and 1.77-fold following α-KG supplementation, respectively. The *gs-gdh* double-silenced strain exhibited 1.68- and 1.57-fold increases, respectively. These results collectively demonstrate that α-KG accumulation enhances the thermotolerance of mycelia and reduces intracellular H_₂_O_₂_ accumulation.

### Heat stress induced reduction in enzymatic activity via degradation of GS protein

**Fig 6:**
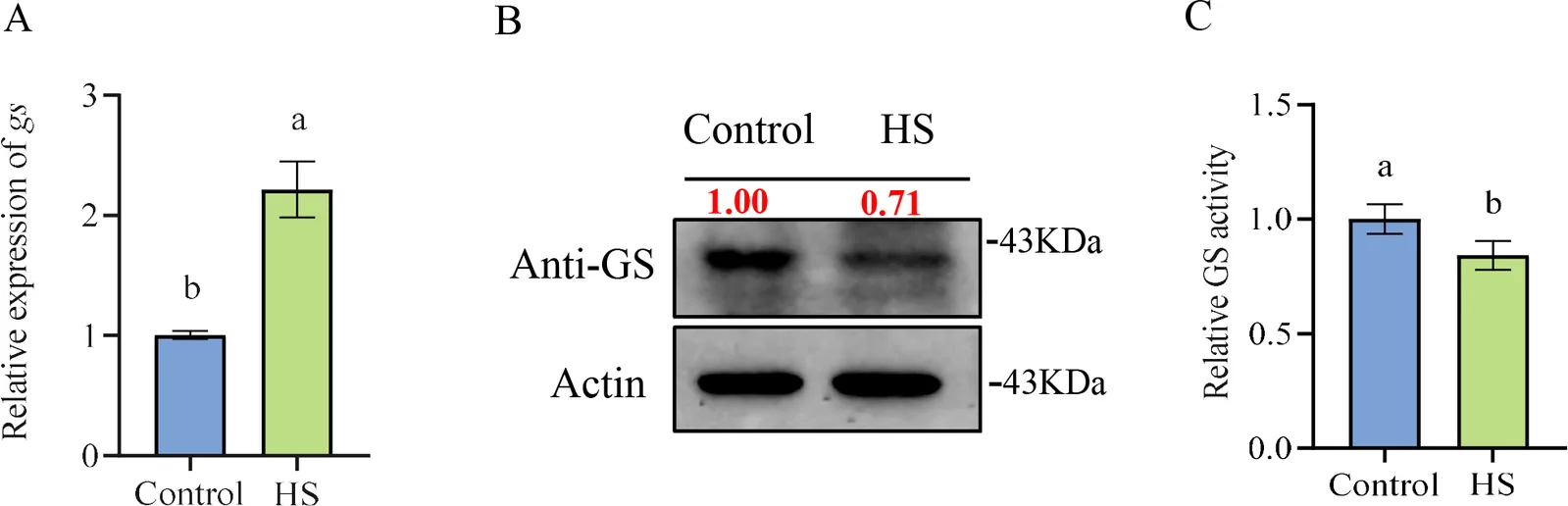
Expression level of the *gs* gene, GS protein level, and enzyme activity. (**A**) Transcription level of *gs* under non-heat and heat stress. The transcription level of *gs* in the WT strain under non-heat stress was defined as 1.0. (**B**) The protein level of GS in the WT strain under non-heat and heat stress was detected by Western blot. The numbers indicate the quantitative determination of protein content using Image v1.8.0 software, and the content of GS protein in the WT strain under normal conditions was taken as the standard. (**C**) GS enzyme activity in the WT strain under non-heat and heat stress. The activity of GS enzyme in the WT strain without heat stress was set to 1. The results are expressed as mean ± standard deviation (*n* = 3). Different letters indicate significant differences between groups (*P* < 0.05, Duncan’s multiple range test).

To investigate the response of GS to heat stress, we first detected the expression of *gs* in WT under both non-heat stress and heat stress. Transcript levels rose 2.21-fold upon heat stress (Fig. 6A). However, immunoblot analysis revealed that heat stress led to a 29.0% decrease in GS protein content (Fig. 6B). Additionally, the corresponding enzymatic activity was significantly reduced by 15.8% (Fig. 6C). Furthermore, we assessed GS protein contents in response to heat stress at varying temperatures (33°C, 37°C, 40°C, and 42°C) and for varying durations at 42°C (10 min, 30 min, 1 h, 2 h, 4 h, and 8 h). Compared with normal conditions (28°C), GS protein contents were significantly reduced as temperatures increased, with decreases of 17.2%, 47.2%, 32.3%, and 50.1%, respectively (Fig. S6A). Under the treatment condition of 42°C, GS protein levels were consistently and significantly lower than the control level at all tested time points, exhibiting a decline with extended exposure duration (Fig. S6B). These data demonstrated that the GS protein content and its activity were reduced under heat stress.

**Fig 7:**
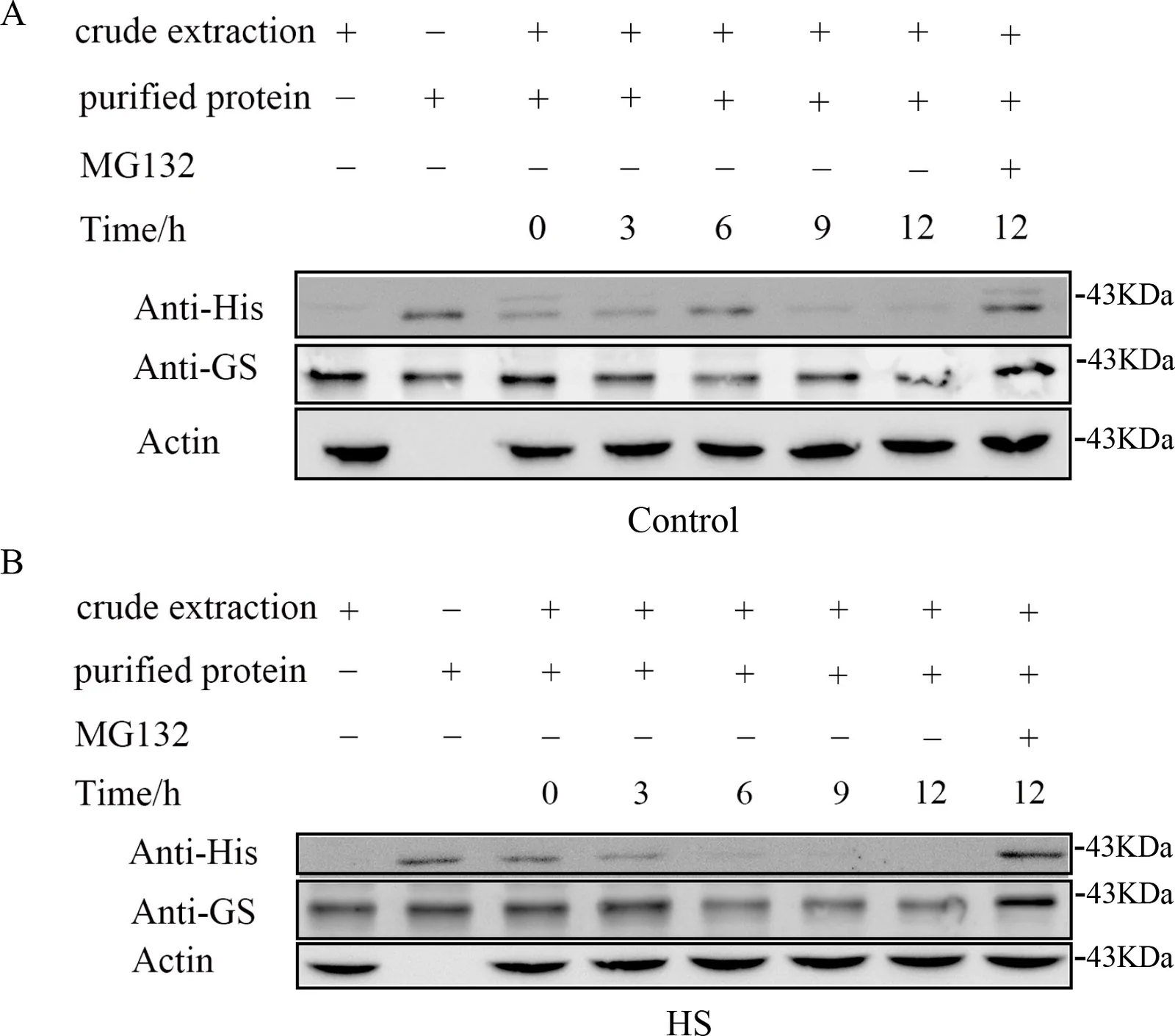
Effects of non-heat stress and heat stress on the stability of GS protein. (**A**) The crude protein extracted from the WT strain under non-heat stress was incubated with GS-His purified protein. The GS protein content was detected by Western blot at different time points. (**B**) The crude protein extracted from the WT strain under heat stress was incubated with GS-His purified protein for different time periods.

To elucidate the mechanisms by which heat stress modulates GS protein content, a cell-free protein degradation assay was conducted to examine the impact of heat stress on GS protein stability. The purified His-tagged GS protein was incubated with crude lysates derived from mycelia cultivated under either normal or heat-stress conditions. When purified GS was exposed to extracts from control mycelia, its abundance declined markedly within 12 h (lane 7, Fig. 7A), whereas co-incubation with the proteasome inhibitor MG132 preserved detectable GS throughout at 12 h (lane 8, Fig. 7A). Under heat stress, the content of GS protein decreased even more rapidly, showing a significant reduction after only 6 h (lane 5, Fig. 7B). Notably, MG132 stabilized GS protein for at least 12 h under heat stress (Fig. 7B). These findings indicate that heat stress accelerated GS protein degradation through a proteasome-dependent pathway.

### Heat stress induced GS degradation via the ubiquitin-proteasome pathway independently of the autophagy-lysosome pathway

A Y2H screen of a cDNA library was performed to identify potential GS-interacting proteins. Among the positive clones, a 20S proteasome β5-type subunit encoded by Gl24873 was screened. The 20S proteasome is the core component of the 26S proteasome, which is responsible for protein degradation (44). The interaction was subsequently validated by Y2H and bimolecular fluorescence complementation assays. For Y2H confirmation, pGBKT7-GS and pGADT7-Gl24873 were co-transformed into the Y2H Gold strain. Transformants grew robustly and formed blue colonies on both low-stringency (SD/−Leu/−Trp) and high-stringency (SD/−Ade/−His/−Leu/−Trp/X-α-Gal) media (Fig. S3A), indicating a specific interaction. In the BiFC assay, co-expression of PVC-GS and PVN-Gl24873 in yeast (SFY2620 strain) produced strong green fluorescence (Fig. S3B). By contrast, all negative-control combinations remained non-fluorescent. These data demonstrated that GS interacted with the proteasomal β5 subunit, supporting the hypothesis that heat-induced degradation of GS was mediated by the 26S proteasome.

**Fig 8:**
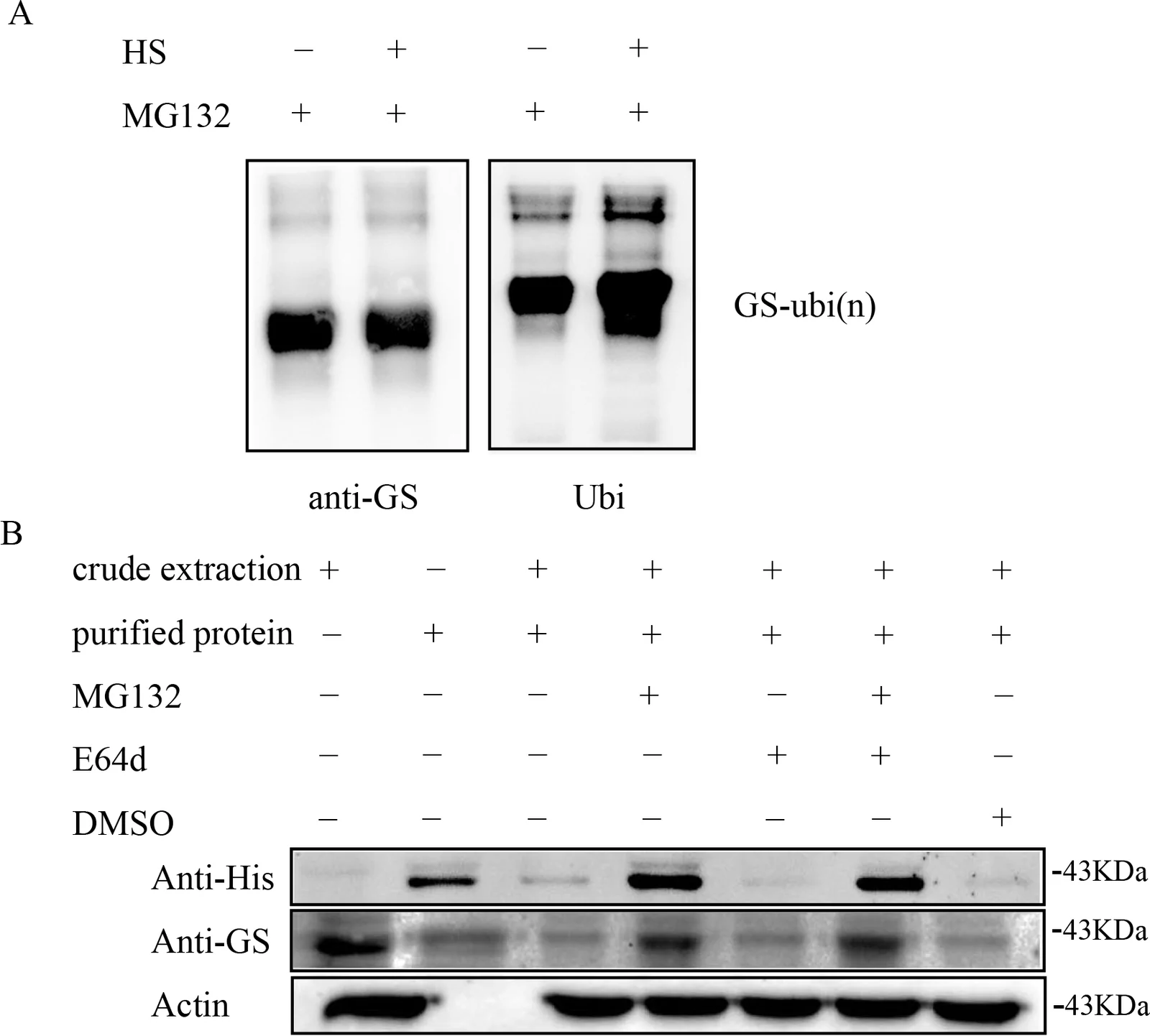
The ubiquitination levels of GS protein and the 26S proteasome degradation pathway. (**A**) The ubiquitination levels of GS protein under non-heat stress and heat stress were detected by ubiquitination assay *in vivo*. (**B**) The crude protein extracted from the WT strain was incubated with GS-His recombinant protein for 12 h to detect the GS protein content. MG132 or E64d was added, respectively, before incubation. DMSO was added as a negative control.

Furthermore, we monitored GS ubiquitination *in vivo* under heat stress. Immunoprecipitation assays revealed an increased level of GS ubiquitination upon heat stress (lane 4, Fig. 8A). Next, to determine whether GS can alternatively be degraded through the autophagy pathway, we established an *in vitro* degradation system combining WT mycelial lysates with recombinant GS-His protein (Fig. 8B). Proteasome activity was blocked with 50 µM MG132, and autophagy-lysosome function was inhibited with 20 µM aloxistatin (E64d). After 12 h of incubation, GS abundance was quantified by immunoblotting. Addition of MG132 alone fully stabilized GS (lane 4 vs lane 3), whereas E64d alone exerted no discernible effect (lane 5 vs lane 3). The effect of co-application of MG132 and E64d was not significantly different from adding MG132 alone (lane 6). No compensatory flux through the autophagy-lysosome pathway was detectable. These data demonstrated that GS degradation was executed via the ubiquitin-proteasome system.

## DISCUSSION

Global warming challenges all organisms to rapidly sense and counteract elevated temperatures. Beyond the well-studied strategies (protein chaperone and antioxidant systems), rapid remodeling of key metabolites, including pyruvate, α-ketoglutarate, trehalose, and sorbitol, is crucial for defense against heat stress (13, 16, 19, 45–47). These metabolites function as compatible solutes, redox buffers, or signaling molecules, playing pivotal roles in heat stress defense (13, 15, 48). Carbon metabolism supplies the carbon skeletons of these key metabolites, along with ATP and the reducing power required to defend against heat stress. Meanwhile, nitrogen metabolism, particularly the glutamate/glutamine hub of the central nitrogen pathway, provides the essential precursors for the *de novo* synthesis of proteins, nucleotides, and all other nitrogen-containing biomolecules that drive growth. Although carbon and nitrogen metabolisms are tightly coupled to support both normal growth and stress responses, their coordinated regulation under heat stress remains unclear.

**Fig 9:**
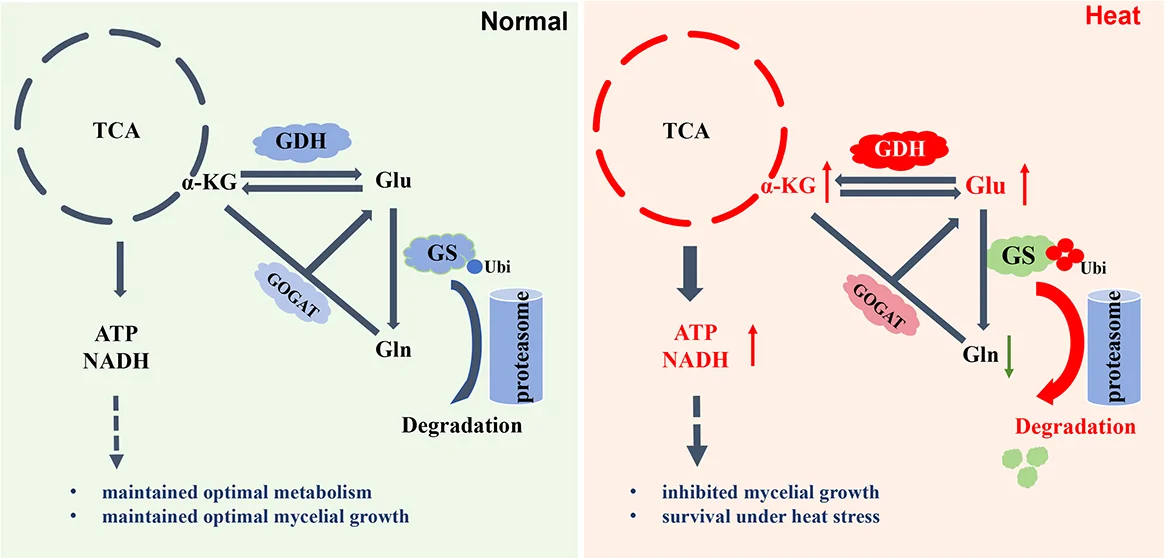
Heat stress reprograms central carbon and nitrogen metabolism through ubiquitin-mediated degradation of glutamine synthetase for survival. Under normal conditions, the TCA cycle functions normally, while the GS-GOGAT cycle sustains glutamine synthesis to support nucleotide and other nitrogenous compound production, maintaining normal mycelial growth. Under heat stress, GS is ubiquitinated and degraded via the 26S proteasome, leading to glutamine deficiency. Concurrently, upregulated GDH drives glutamate conversion to α-ketoglutarate, fueling enhanced TCA cycle activity and increased ATP/NADH production, thus contributing to mycelial survival under heat stress.

Here, using the basidiomycete *Ganoderma lucidum* as a model, we demonstrated that heat stress reprograms central carbon/nitrogen metabolism through ubiquitin-mediated degradation of glutamine synthetase. Upon temperature rise, GS was polyubiquitylated and degraded by the 26S proteasome, leading to a rapid decline in GS activity and an accumulation of glutamate. The elevated glutamate was then channeled into the TCA cycle via GDH. This metabolic rerouting replenished α-ketoglutarate, enhanced ATP and NADH production, and ultimately supported fungal growth under heat stress (Fig. 9). Our findings revealed that a ubiquitin-dependent signal instantaneously rebalances central carbon-nitrogen metabolism under heat stress, offering a new mechanistic perspective on a direct and rapid link between central metabolism and stress defense.

Glutamine synthetase is a pivotal enzyme in central nitrogen metabolism, catalyzing the ATP-dependent conversion of glutamate to glutamine. As the primary donor of amino groups, glutamine serves as an essential precursor for the biosynthesis of nucleotides, cofactors, and multiple amino acids, thereby exerting broad control over fungal nitrogen metabolism, growth, and development. Loss of function of the *gs*-encoding protein in *Magnaporthe oryzae*, *Aspergillus flavus*, and arbuscular mycorrhizal fungi consistently reduced intracellular glutamine pools, leading to pleiotropic defects in hyphal growth, secondary metabolism, and virulence (25, 49, 50). Our previous study also demonstrated that glutamine synthetase was the key enzyme for glutamine biosynthesis in *Ganoderma lucidum*, influencing hyphal growth and ganoderic acid production; its activity was regulated by the central nitrogen-responsive transcription factor AreA (24). Reports have documented that in plants, the activity of GS was diminished under salt or drought stress (51–53). However, the functional role of fungal GS in defending against stresses remains largely uncharacterized. In our study, heat stress suppresses GS activity, which in turn upregulated the nitrogen-metabolic enzyme glutamate dehydrogenase and redirected glutamate flux toward α-KG. α-KG functions not only as a metabolic node linking carbon and nitrogen assimilation but also participates in diverse biological processes, including antioxidative defense, signaling cascades, and epigenetic regulation (54). Here, we additionally demonstrated that exogenous α-KG supplementation markedly attenuated heat stress-induced mycelial growth inhibition and reduced intracellular H_₂_O_₂_ accumulation. The heat-induced accumulation of α-KG suggested an adaptive strategy for survival under heat stress. We further confirmed that GS degradation occurred across a physiologically relevant temperature range (33°C−42°C). These findings suggest that proteasome-mediated protein degradation participates in metabolic adaptation to heat stress even at moderate temperatures, extending beyond its canonical role in clearing misfolded proteins under extreme conditions.

Multiple modes of post-translational modification (PTM) that tune carbon metabolism have been studied. In cancer cells, key glycolytic enzymes, including phosphoglycerate kinase 1 and 6-phosphofructo-2-kinase/fructose-2,6-bisphosphatase 3, were acetylated, thereby intensifying glycolytic flux to meet the bioenergetic and biosynthetic demands of rapid proliferation (55, 56). Protein phosphorylation plays a role in glycolysis through the regulation of enzyme activity in ovine muscle (57, 58). In *Saccharomyces cerevisiae*, several glycolytic enzymes, including the 6-phosphofructo-2-kinase (Pfk26), the phosphofructokinases (Pfk1 and Pfk2), and the pyruvate kinase Cdc19, serve as PTM hotspots where phosphorylation, acetylation, and ubiquitination converge, thereby functioning as pivotal regulatory nodes that integrate diverse signals to control carbon flux (59, 60). After heat stress, deacetylase GlSIRT1 increases pyruvate kinase activity through deacetylation, thereby increasing pyruvate content in *G. lucidum* (61). In contrast, post-translational control of nitrogen-metabolic enzymes remains sparsely documented. In bacteria, adenylylation/deadenylylation serves as a canonical mechanism for rapidly inactivating/reactivating glutamine synthetase activity (62). We previously demonstrated that GSNOR responds to heat stress and regulates secondary metabolite content via protein S-nitrosylation (33). NO-dependent nitrification suppresses GS activity during nitrate assimilation, although whether a denitration mechanism exists remains unresolved (24). However, post-translational modifications of GS, particularly ubiquitination, have not been explicitly reported in fungi. The present study establishes that polyubiquitylation triggers 26S-proteasomal degradation of glutamine synthetase, resulting in a rapid drop in glutamine synthetase activity under heat stress. Nevertheless, the precise molecular mechanism underlying GS degradation remains elusive. Although NO-mediated nitration has been reported to suppress GS activity, the potential crosstalk between S-nitrosylation and ubiquitination has yet to be investigated. Furthermore, the specific E3 ubiquitin ligase targeting GS under heat stress has not been identified, constituting a critical gap in our mechanistic understanding. Future studies utilizing proteomics or yeast two-hybrid screening could identify specific E3 ligases mediating GS ubiquitination, thereby refining the molecular mechanism of GS metabolic regulation under heat stress.

The ubiquitin-proteasome system and the autophagy-lysosome pathway (ALP) constitute the two principal routes for protein degradation. By dictating protein stability, they exert pervasive control over diverse physiological processes. Ubiquitin-mediated proteolysis not only sets the abundance of key regulatory proteins and enzymes but also plays a pivotal role in abiotic stress responses (63, 64). Likewise, the ALP coordinates growth and stress adaptation under various adverse conditions (65). Notably, in plants, the UPS has been shown to reprogram metabolism by degrading key metabolic enzymes in response to salt and drought stress (66, 67). However, research on the role of the ubiquitin-proteasome system in metabolic reprogramming in response to abiotic stress remains poorly characterized in fungi. Here, we demonstrate that heat-triggered degradation of glutamine synthetase proceeds via the UPS rather than the ALP. Such UPS-dependent removal of a key nitrogen-metabolic enzyme allows the fungus to rapidly readjust carbon-nitrogen balance and reallocate energy and reducing power in favor of survival. Determining whether this UPS-mediated metabolic switch represents a conserved stress-adaptation strategy across fungi requires phylogenetically broader evidence. Future investigations should therefore assess the ubiquitination status of GS and its 26S proteasome-dependent degradation under heat stress across a broader phylogenetic spectrum, including additional basidiomycetes (e.g., *Lentinula edodes* and *Pleurotus ostreatus*) and ascomycetes. Such comparative analyses would be instrumental in elucidating the phylogenetic conservation of this regulatory pathway.

## Data Availability

The data that support the findings of this study are available from the corresponding author upon request.

## SUPPLEMENTAL MATERIAL

Supplemental material aem.00602-26-s0001.docx Fig. S1 to S13; Table S1.

ASM does not own the copyrights to Supplemental Material that may be linked to, or accessed through, an article. The authors have granted ASM a non-exclusive, world-wide license to publish the Supplemental Material files. Please contact the corresponding author directly for reuse.
