# Supplementary material for: Heat stress-induced degradation of glutamine synthetase rebalances central carbon-nitrogen metabolism and promotes thermotolerance in Ganoderma lucidum
Source: Appl Environ Microbiol. 2026 Jul 21;92(8):e00602-26. doi: 10.1128/aem.00602-26 (PMC13488339; doi:10.1128/aem.00602-26)
Supplement: Supplementary file 1 [file aem.00602-26-s0001.docx]

Supplementary materials for

**Heat Stress Induced Degradation of Glutamine Synthetase Rebalances Central Carbon-Nitrogen Metabolism and Promotes Thermotolerance in *Ganoderma lucidum***

Jinjin Qiao^1^, Huajun Li^1^, Yuzhen Yang^1^, Yanqiu Li^1^, Shangshang Zhang^1^, Mingwen Zhao^*1^, Jing Zhu^*1^.

1 Department of Microbiology, College of Life Sciences, Nanjing Agricultural University, Nanjing 210095, Jiangsu, P.R. China.

Affiliations: Department of Microbiology, College of Life Sciences, Nanjing Agricultural University, Nanjing 210095, Jiangsu, P.R. China.

*Corresponding author: Mingwen Zhao, Jing Zhu

E-mail: [mwzhao@njau.edu.cn](mailto:mwzhao@njau.edu.cn), [jingzhu@njau.edu.cn](mailto:jingzhu@njau.edu.cn)

Tel: +0086-25-84395602

**Fig. S1** The effects of MSX on mycelial growth and GS activity. **A**. Mycelial growth of WT strains in different concentrations of MSX. **B**. The diameter of the WT strains under different concentrations of MSX. **C**. Relative inhibition rate of mycelial growth of WT strains in different concentrations of MSX. The relative inhibition rate was calculated as (Dc-Dt) /Dc×100 (%), where Dc and Dt represent the mycelial diameter without adding MSX and the mycelial diameter with different concentrations of MSX, respectively. **D**. The GS enzyme activity of WT strains was detected at different MSX concentrations (0 mM, 1 mM, 1.5 mM, 2 mM, 2.5 mM and 3 mM). The activity of GS enzyme in the WT strains treated without MSX was set to 1. Results are expressed as mean ± SD (n = 3). Different letters indicated significant difference between groups (*p* < 0.05, Duncan's multiple range test).

**Fig. S2** The effects of R162 on mycelial growth and GDH activity, and the construction of gs-silenced strains and *gs*-*gdh* co-silenced strains. **A**. Mycelial growth of the WT strain in different concentrations of R162 (0 mg·mL⁻¹, 0.005 mg·mL⁻¹, 0.01 mg·mL⁻¹, 0.02 mg·mL⁻¹, 0.03 mg·mL⁻¹, and 0.04 mg·mL⁻¹, respectively). **B**. Colony diameter of the WT strain in different concentrations of R162. **C**. Relative enzyme activity of GDH in the WT strain at different concentrations of R162. The enzyme activity of GDH in the WT strain without heat stress treatment were set to 1. **D**. The expression levels of *gdh* in WT, CK, and *gdh*-silenced strains. **E**. The expression levels of *gs* and *gdh* in WT, CK, and *gs*-*gdh* co-silenced strains. Results are expressed as mean ± SD (n = 3). Different letters indicate significant differences between groups (*p*< 0.05, Duncan's multiple range test).

**Fig. S3** GS interacts with the Gl24873. **A**. pGBKT7-GS and pGADT7- Gl24873 were co-transformed into the Y2H strain. The Y2H strain containing the target vector was cultured at 30℃ for 2 days on two synthetic dropout media (SD/Leu/-Trp) and (SD/-Ade/-His/-Leu/-Trp) with 20 ng/mL X-α-gal and 125 ng/mL AbA. The strain with pGBKT7-53 and pGADT7-T was used as the positive control. The strain with pGBKT7-Lam and pGADT7-T was used as the negative control. **B**. Bimolecular fluorescence complementary assay indicated the interaction between GS and proteasome Gl24873. PVN-Gl24873 and PVC-GS vectors containing GFP tags were constructed and transformed into yeast cells to detect fluorescence phenomena. DIC represents yeast cell morphology under normal white visual field, Venus represents yeast cell morphology under green fluorescence. Scale bar represents 0.03 mm.

**Fig.S4.** Effects of exogenous supplementation of different concentrations of α-KG on mycelial growth. A. Mycelial growth of the wild-type strain under different α-KG concentrations (0, 30, 60, 90, 120 and 150 mg L⁻¹) under non-heat stress and heat stress conditions; B. The relative inhibition rate of the wild-type strain with exogenous addition of different concentrations of α-KG under non-heat stress and heat stress conditions was calculated using the formula (Dc-Dt)/Dc × 100%, where Dc and Dt represent the colony diameters under non-heat stress and heat stress conditions, respectively. The results are expressed as mean ± standard deviation (n=3). Different letters indicate significant differences between groups (*p*<0.05, Duncan's multiple range test).

**Fig.S5.** Effects of exogenous α-KG supplementation on mycelial growth and intracellular H_2_O_2_ content in *G. lucidum*. A. Mycelial growth of the wild type (WT), control (CK), and exogenous α-KG (60 mg L⁻¹) supplemented WT, CK, *gdh*-silenced strain, and *gs*-*gdh* double-silenced strain under non-heat stress and heat stress conditions. B. The relative inhibition rate of different strains under non-heat stress and heat stress was calculated using the formula (Dc-Dt)/Dc × 100%, where Dc and Dt represent the colony diameters under non-heat stress and heat stress conditions, respectively. C. H₂O₂ content in different strains under non-heat stress and heat stress conditions. Relative H₂O₂ content was calculated as the ratio of H₂O₂ content in each strain to that in the wild-type strain under non-heat stress conditions. Error bars indicate the standard deviation of the mean from three replicate experiments. Data are presented as mean ± SD (n = 3). Different letters indicate significant differences among groups (*p* < 0.05, Duncan's multiple range test).

**Fig.S6.** Effects of different temperatures and treatment durations on GS protein content. A. GS protein levels in the wild-type strain under different temperatures (33°C, 37°C, 40°C, and 42°C) were detected by Western blotting. The values represent quantitative protein content determinations performed using ImageJ v1.8.0 software, with GS protein content in the wild-type strain under normal conditions serving as the standard value. B. GS protein levels in the wild-type strain treated at 42°C for different durations (10 min, 30 min, 1 h, 2 h, 4 h, and 8 h) were detected by Western blotting. The values represent quantitative protein content determinations performed using ImageJ v1.8.0 software, with GS protein content at 0 min serving as the standard value.

**Source data for Figure 6B**

**Fig. S7** Uncropped Western blots

**Source data for Figure 7A**

**Fig. S8** Uncropped Western blots

**Source data for Figure 7B**

**Fig. S9** Uncropped Western blots

**Source data for Figure 8A**

**Fig. S10** Uncropped Western blots

**Source data for Figure 8B**

**Fig. S11** Uncropped Western blots

**Source data for Figure S6A**

**Fig. S12** Uncropped Western blots

**Source data for Figure S6B**

**Fig. S13** Uncropped Western blots

**Table S1** Primers used in the experiment

| **Primers** | **Primer sequences (5′ to 3′)** | **Description** |
| --- | --- | --- |
| RT-18S-F | TATCGAGTTCTGACTGGGTTGT | Detects the *18S* expression |
| RT-18S-R | ATCCGTTGCTGAAAGTTGTAT |  |
| RT-GS-F | ACCAACTTCCGCCACCAT | Detects the *gs* expression |
| RT-GS-R | AAGACCTTGCCAGCACCAG |  |
| RT-GDH-F | GAGCAGCGAGCAAAGGT | Detects the *gdh* expression |
| RT-GDH-R | CGATGATGTGGTCGGAGAT |  |
| *gdh*i-F | actcttcatccccctggtaccATGTCCACCCCACCGAGC | For the construction of *gdh* silenced vector |
| *gdh*i-R | gcgcacaggcggagaactagtGATGATGTGGTCGGAGATGACC |  |
| *gs-gdh*i-F | actcttcatccccctggtaccATGGCTCAGCAGTACCGCC | For the construction of *gs-gdh* double-silenced vector |
| *gs-gdh*i-R | gcgcacaggcggagaactagtGATGATGTGGTCGGAGATGACC |  |
| pGBKT7-GS-F | ATGGCTCAGCAGTACCGCC | For cloning the *gs* gene into the pGBKT7 vector |
| pGBKT7-GS-R | TCAGCGCACGTCGTATGTGA |  |
| pGADT7- Gl24873-F | ATGAACACCTTCTTGACTCGCTAT | For cloning the *Gl24873* gene into the pGADT7 vector |
| pGADT7- Gl24873-R | TTATGCCTCTCCTTCACTTTGCG |  |
| GS-PVC-F | aaggccgctgcagctactagtATGGCTCAGCAGTACCGCC | For cloning the *gs* gene into the PVC vector |
| GS-PVC-R | gataagcttgatatcgaattcTCAGCGCACGTCGTATGTGA |  |
| Gl24873-PVN-F | gctgcagctactagtggatccATGAACACCTTCTTGACTCGCTATT | For cloning the *Gl24873* gene into the PVN vector |
| Gl24873-PVN-R | gataagcttgatatcgaattcTTATGCCTCTCCTTCACTTTGCG |  |
